# Presenteeism and mental health of workers during the COVID-19 pandemic: a systematic review

**DOI:** 10.3389/fpubh.2023.1224332

**Published:** 2023-09-14

**Authors:** Juan Jesús García-Iglesias, Juan Gómez-Salgado, Joao Apostolo, Rogério Rodrigues, Emília Isabel Costa, Carlos Ruiz-Frutos, Santiago Martínez-Isasi, Daniel Fernández-García, Ángel Vilches-Arenas

**Affiliations:** ^1^Sociology, Social Work and Public Health, Faculty of Labour Sciences, University of Huelva, Huelva, Spain; ^2^Health Sciences Research Unit: Nursing (UICISA: E), Coimbra Nursing School, Coimbra, Portugal; ^3^Escuela de Posgrado, Universidad de Especialidades Espíritu Santo, Guayaquil, Guayas, Ecuador; ^4^Nursing Department, Health School, University of Algarve, Faro, Portugal; ^5^Simulation and Intensive Care Unit of Santiago (SICRUS), Health Research Institute of Santiago de Compostela (IDIS), Santiago de Compostela, Galicia, Spain; ^6^CLINURSID Research Group, Faculty of Nursing, University of Santiago de Compostela, Santiago de Compostela, Galicia, Spain,; ^7^Health Research Nursing Group (GREIS), Department of Nursing and Physiotherapy, University of Leon, Leon, Spain; ^8^Preventive Medicine and Public Health, University of Seville, Seville, Spain; ^9^Preventive Medicine, Virgen Macarena University Hospital, Seville, Spain

**Keywords:** COVID-19, mental health, occupational health, presenteeism, workers

## Abstract

**Background:**

A large number of workers attend work despite being ill. Attending work during sickness can have a number of consequences for the worker (e.g., worsening of physical and mental condition), for co-workers, and for the company, and for service users.

**Objectives:**

The aim of this study was to assess the factors influencing presenteeism and mental health of workers during the COVID-19 pandemic.

**Methods:**

A systematic review following the PRISMA format was conducted in the PubMed, Scopus, Web of Science (WoS), Cumulative Index to Nursing and Allied Health Literature (CINAHL), PsycInfo, and ScienceDirect electronic databases in January 2023, using the following key words: Presenteeism, Mental Health, and COVID-19. The eligibility criteria applied were original articles published in English, Spanish, French, German, and Portuguese, workers during the COVID-19 pandemic (data collection date: January 01, 2020 – January 01, 2023), and articles assessing at least one measure of presenteeism and mental health status. Methodological quality was assessed using the critical appraisal tools of the Joanna Briggs Institute. The followed protocol is listed in the International Prospective Register of Systematic Reviews (PROSPERO) with code CRD42023391409.

**Results:**

A total of 25 studies were included in this review recruiting a total of 164,274 participants. A number of factors influencing mental health and sickness presenteeism were identified: (1) mental health-related factors (burnout [in 4 studies], stress [in 9 studies], depression [in 1 study], fear of COVID-19 [in 1 study], no well-being [in 2 studies], etc.); (2) individual factors (health status [in 1 study], being young [in 1 study], workers who experienced interrupted medical care [in 2 studies], having a chronic disease [in 1 study], etc.); (3) factors related to the situation caused by COVID-19 (confinement, symptoms, loss of contract, risk of bankruptcy, etc. [in 1 study each one]); and (4) factors derived from working conditions (organisational support [in 1 study], patient care [in 1 study], work functioning or task performance impairment [in 4 studies], work fatigue [in 2 studies], safety climate [in 1 study], workload [in 1 study], etc.).

**Conclusion:**

Identifying the key determinants of presenteeism and understanding the phenomena and origins of sickness presenteeism will help to create a safe working environment and optimal organisational systems to protect vulnerable workers in a pandemic context.

**Systematic review registration:**

The unique identifier is CRD42023391409.

## 1. Introduction

Work attendance during illness can be an occupational health and public health problem, as it is directly related to productivity and the worker’s perception of ineffectiveness (1). Sickness presenteeism is a type of behaviour displayed by some workers who, despite being ill and having physical and/or psychological conditions, decide to go to work or continue with their workday (2). On the other hand, presenteeism means that workers attend work physically and comply with their working hours, but do not really work or contribute anything beyond their presence (3). It is estimated that around 1 in 3 European workers engage in sickness presenteeism (3) and the cost that presenteeism incurs in the workplace is higher than the cost of treatment for these physical and mental illnesses, or even absenteeism (failing to work due to sickness) and sick leaves (4).

This decision is usually made autonomously by the worker and may depend on personal characteristics, the economic situation and type of work, the individual’s values and concerns about leaving their job unattended, among other things (5). In fact, both job demands and resources may be elements that influence a worker’s decision to work despite being sick, according to the Job Demands-Resources Model (6, 7). In this line, authors such as Pohling et al. (8) focused on occupational environmental factors and work climate as the theoretical basis for explaining presenteeism according to the Person-Environment (mis)fit theory. When a misfit between work and the person occurs as a result of these factors, workers experience stress and subsequent psychological burnout as a result. This misfit, together with the need to save resources, leads to workers continuing to work despite this situation (9).

Attending work during sickness can have a number of consequences for the worker (e.g., worsening physical and mental condition), for co-workers, and for the company and service users (10), hence the importance of its study and evaluation. Nevertheless, before the pandemic, some authors had already found possible links between certain mental health-related problems (such as depression) and a change in productivity caused by sickness presenteeism (11–13).

According to the Cambridge dictionary (14), a worker is someone who works in a particular job or in a particular way or someone who works for a company or organisation but does not have a powerful position. Self-employed workers or workers in small businesses may have replacement difficulties in the event of absence from work and are often compelled to work despite being ill (15). A study in Portugal determined that self-employed workers were 85% less likely to take sick leave than employees (14). This can be justified by the need to continue working despite being sick because of the economic difficulties in general, and the pandemic in particular, in order to find solutions to keep their businesses going (16). Another occupational group with high levels of sickness presenteeism is healthcare workers (5). In this case, work attendance during illness can undermine the quality of care provided, increase the likelihood of incidents that may compromise patient safety and clinical practise (17), and even lead to infecting patients and/or co-workers (18). Specific factors justifying these high levels of presenteeism or sickness presenteeism among health workers may include feelings of professionalism and loyalty (19), personal circumstances, and working conditions (stressful work, high complexity, long working hours, low staffing levels, etc.) (18, 20), situations that worsened considerably during the COVID-19 pandemic.

The COVID-19 pandemic has led many organisations to change the way they work and, consequently, the working conditions of their workers. In addition to the pandemic’s impact on people’s mental health (21), the economic situation and job insecurity also worsened (some people worked despite being ill in order not to lose their jobs), some workers switched to teleworking (teleworking from home despite being ill and performing work duties outside of their working hours), chronic programmes were temporarily suspended, and people’s health and consumption habits began to change, with the subsequent consequences at the physical, mental, and social levels (22). Despite recommendations for social distancing and isolation in the case of COVID-19-like symptomatology, many workers were forced to work in order not to lose their jobs or see their income reduced, especially workers with a lower level of education and lower socio-economic status (23), with the consequent impact this may have on their mental health. This study is necessary so as to know the factors that influence presenteeism and to assess which professions suffer the most from sickness-related presenteeism so that organisations and/or companies can take measures based on scientific evidence.

Therefore, the aim of this study was to assess the factors associated with mental health and working conditions that affect presenteeism of workers during the COVID-19 pandemic.

## 2. Methods

### 2.1. Study design

A systematic review of association (aetiology and risk) (24) was conducted following the PRISMA statement guidelines (Preferred Reporting Items for Systematic reviews and Meta-Analyses) (25, 26). The followed protocol is listed in the International Prospective Register of Systematic Reviews (PROSPERO) with code CRD42023391409. This topic was not covered recently by a Review in IJPH or in another journal.

### 2.2. Databases and search strategy

The search was carried out in the Pubmed, Scopus, Web of Science (WoS), Cumulative Index to Nursing and Allied Health Literature (CINAHL), PsycInfo, and ScienceDirect electronic databases based on the keywords that the research question yielded following the PEO strategy (27). The research question was *What are the factors related to mental health and working conditions that affect presenteeism of workers during the COVID-19 pandemic?* (Table 1).

**Table 1:** PEO format: keywords.

Population	Workers during the COVID-19 pandemic
Exposure	Factors related with mental health and working conditions
Outcomes	Presenteeism: prevalence of presenteeism; consequences and main manifestations; short/medium/long-term effects; influence of mental health on presenteeism; differences between countries and professions; other factors associated with presenteeism; differences between telework and face-to-face work; and possible causes of presenteeism.
**Research question**	
What are the factors related to mental health and working conditions that affect presenteeism of workers during the COVID-19 pandemic?	

Following these keywords, the Medical Subject Headings (MeSH) thesaurus was consulted, yielding the descriptors Presenteeism, Mental Health, and COVID-19. In order to improve the collection of published studies in line with the subject of the study, synonymous terms were used to complete the search strategy based on the MeSH descriptors (Table 2), which were joined using the Boolean operators *and* and *or*.

**Table 2:** Terms and definitions used in the search.

MeSH terms	Meaning	Terms
Presenteeism	Reporting for work despite feeling ill	Presenteeism OR sickness presence
Mental Health	Emotional, psychological, and social well-being of an individual or group	Mental Health OR Burnout OR Stress OR Anxiety OR Depression
COVID-19	A viral disorder generally characterised by high fever; cough; dyspnoea; chills; persistent tremor; muscle pain; headache; sore throat; a new loss of taste and/or smell (see ageusia and anosmia); and other symptoms of a viral pneumonia	COVID-19 *OR* SARS-CoV-2

MeSH, Medical Subject Headings.

In this case, the terms *Presenteeism, sickness presence, Mental Health, Burnout, Stress, Anxiety, Depression, COVID-19,* and *SARS-CoV-2* were used. Table 3 shows the search strategy used, carried out on January 17, 2023, for each of the aforementioned databases during the search process.

**Table 3:** Search strategy used in each database.

Databases	Search strategy
PubMed	(“presenteeism”[Title/Abstract] OR “sickness presence”[Title/Abstract]) AND (“COVID-19”[Title/Abstract] OR “SARS-CoV-2”[Title/Abstract]) AND (“mental health”[Title/Abstract] OR “burnout”[Title/Abstract] OR “stress”[Title/Abstract] OR “anxiety”[Title/Abstract] OR “depression”[Title/Abstract])
Scopus	(TITLE-ABS-KEY (mental AND health OR burnout OR stress OR anxiety OR depression) AND TITLE-ABS-KEY (presenteeism OR sickness AND presence) AND TITLE-ABS-KEY (covid-19 OR sars-cov-2))
Web of Science	“mental health” OR burnout OR stress OR anxiety OR depression (Topic) AND presenteeism OR sickness presence (Topic) AND COVID-19 OR SARS-CoV-2 (Topic)
CINAHL	AB (“mental health” OR burnout OR stress OR anxiety OR depression) AND AB (presenteeism OR sickness presence) AND AB (COVID-19 OR SARS-CoV-2)
PsycInfo	tiab(mental health OR burnout OR stress OR anxiety OR depression) AND tiab(presenteeism OR sickness presence) AND tiab(COVID-19 OR SARS-CoV-2)
ScienceDirect	Title, abstract, keywords: (mental health OR burnout OR stress OR anxiety OR depression) AND (presenteeism OR sickness presence) AND (COVID-19 OR SARS-CoV-2)
Other sources	Items identified through other resources
Search date: January 17, 2023	

### 2.3. Selection criteria

The following criteria were used for the selection of articles:

#### 2.3.1. Inclusion criteria

- Original articles published in English, Spanish, French, German, and Portuguese.
- Type: original articles, short communications, and case reports.
- Population: workers during the COVID-19 pandemic (someone who works in a particular job or in a particular way or someone who works for a company or organisation).
- Data collection date: January 01, 2020 – January 01, 2023.
- Articles assessing any of the following values and/or effects and those that include at least one measure of presenteeism and mental health status (presenteeism, mental health, and factors associated): prevalence of presenteeism or sickness presenteeism, consequences and main manifestations, short/medium/long-term effects; influence of mental health on sickness presenteeism, and possible causes of sickness presenteeism; and other factors that reduce or increase presenteeism or sickness presenteeism; differences between countries and professions; differences between telework and face-to-face work.

#### 2.3.2. Exclusion criteria

- Studies of low scientific-technical quality after applying the quality assessment tool.
- Population: students.
- Date of data collection: if out of the inclusion period or if the date of data collection was missing.
- Articles that did not answer the research question and were not related to the objective of the review. Studies that did not assess presenteeism or sickness presenteeism as well as mental health were excluded.
- Type: opinion articles, editorials, and letters to the editor.

### 2.4. Data collection and extraction

Based on the aforementioned consensual search strategy, two investigators independently performed the searches, eliminated duplicate studies, and selected articles for inclusion after reading the abstract and title according to the previously established criteria. Subsequently, the same two authors reviewed the full text of potentially eligible studies and the decision to include or exclude them in the review was made by consensus. Discrepancies were resolved by a third author. For the data collection after reading the full text of the articles, specific information on the studies was extracted, such as authors’ names and year of publication; context in which the study was conducted; objective of the study; type of study; sample, methodology, and instruments used for data collection; main findings; and quality of the study after applying the critical appraisal tools.

### 2.5. Methodological quality assessment

Two reviewers independently determined the methodological quality of the selected studies using the critical appraisal tools of the Joanna Briggs Institute (JBI) at the University of Adelaide. These tools allowed assessing the methodological quality of a study and determining the extent to which a study has excluded or minimised the risk of bias in its design, conduct, and/or analysis. The versions for analytical cross-sectional studies (8-items) (28), for qualitative research (10 items) (29), for cohort studies (11 items), and for Randomised Controlled Trials (13 items) (30) were used, setting the cut-off point at 6 to be accepted for inclusion in this review for the first two, 8 for the third, and 10 for the fourth (Supplementary material). The basic parameters of the included articles conform to the applied inclusion criteria (especially study design, year of publication, and country origin).

## 3. Results

**Figure 1:**
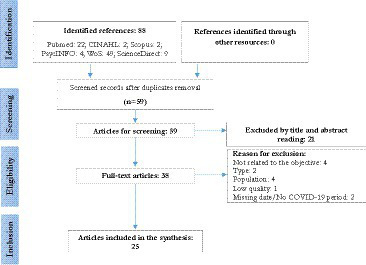
Search results (PRISMA – Flowchart).

The initial search strategies identified a total of 88 references, which were then screened according to the topic of this review. A total of 25 studies were finally selected (Figure 1), recruiting a total of 164,274 participants. 22 of which were analytical cross-sectional studies, 1 qualitative research, 1 cohort study, and 1 randomised controlled trial.

Four studies had been conducted in Japan (31–34) and 4 in United Kingdom (35–38), 3 in United States (39–41), 2 in China (42, 43), Germany (44, 45), and the Republic of Korea (46, 47), and 1 in Sweden (16), Wales (48), Canada (22), Turkey (49), Lithuania (50), Portugal (51), Australia and New Zealand (52), and Belgium and the Netherlands (53). In 14 of the 25 selected articles, collection took place in 2020; 6 of the 25 were collected in 2021; and the remaining 5 were collected over months in both 2020 and 2021. No studies were found with data collected in 2022 or later. Regarding participants, in 10 studies the sample consisted of health professionals, in 2 studies the sample was collected in the educational environment, and another 2 samples included self-employed workers. The remaining studies (11 out of 25) included workers from other occupational fields or general workers. Working from home or remote working was assessed in 3 of the 25 studies.

It was found that between 70.6% (43) and 26% (38) of the subjects in the included studies showed sickness presenteeism. In addition, a number of factors may have also favoured presenteeism or sickness presenteeism, such as mental health-related factors [burnout (34, 45, 49, 51), stress (33–35, 38, 42, 44, 45, 47, 49), depression (46), fear of COVID-19 (49), no well-being (16, 40), cyberbullying (51), sleep disturbance (34), concern about having enough food (41), social isolation (38), and no resilience (38)]; individual factors [poor marital relationship (31), health status (42), being young (38), attention-deficit/hyperactivity disorder symptoms (32), workers who experienced interrupted medical care (33, 40), low physical activity (38, 50), sedentary behaviours (52), having children (41), having health insurance (41), and having a chronic illness (38)]; factors related to the situation caused by COVID-19 [confinement (45), having symptoms of respiratory infectious disease (48), not volunteering to work on the frontline (47), impact on business operations, loss of contract, and risk of bankruptcy (16)]; and factors arising from working conditions [perceived organisational support (49), direct patient care (39), work functioning or task performance impairment (31, 42, 43, 53), work fatigue (34, 43), safety climate (22), workload (22), having no one to replace them (48), geographical distribution (48), transition from in-person to online modes of working (34, 35), salary of less than $35,000 (41), increase in working hours, work–family conflict (16)].

The included studies were assessed with the JBI critical appraisal tool, where analytical cross-sectional studies, qualitative research, and randomised controlled trials obtained medium-high scores.

Table 4 shows the characteristics of each of the 25 studies included in this review.

**Table 4:** Characteristics of the studies included in the systematic review.

Studies	Context	Study objective	Type of study	Participants	Methods	Main findings	JBI
Basar et al. (51)	Turkey May–June 2021	To uncover whether nurses’ fear of contracting COVID-19 has resulted in stress-related presenteeism and burnout, and whether perceived organisational support is effective in dealing with both nurses’ fear of contracting COVID-19 and its undesired consequences.	Cross-sectional study	513 Nurses	- Stress-related Presenteeism Scale - Perceived Organisational Support Scale - Fear of COVID-19 Scale - Burnout Scale	They reported notable levels of burnout (M = 4.51, SD = 1.47) and stress-related presenteeism (M = 3.29, SD = 1.01), as well as slightly inadequate levels of perceived organisational support (M = 2.30, SD = 1.07). Fear of COVID-19 infection resulted in burnout (*β* = 0.35, *p* < 0.001) and stress-related presenteeism (*β* = 0.39, *p* < 0.001). Stress-related presenteeism also resulted in burnout (*β* = 0.50, *p* < 0.001), mediating the relationship between fear of contracting COVID-19 and burnout.	8/8
Cheslack-Postava et al. (41)	United States April–June 2020	To assess occupational circumstances associated with adverse mental health among health care workers during the COVID-19 pandemic.	Cross-sectional study	2,076 HCWs	- PHQ-9 - GAD-7 2nd outcomes: COVID-related occupational experiences, stress, and anger	50% of the population experienced symptoms, but did not work while sick, 15% worked while sick but not in direct patient care, and 35% worked in direct patient care while sick. Presenteeism experiences were associated with OR of negative mental health of the following: 1.28 (0.98–1.67), *p* = 0.07 for those with symptoms who did not work; 1.48 (0.99–2.22), *p* = 0.05 for those who worked while sick but not in direct patient care; and 2.29 (1.71–3.08), *p* < 0.001 for those who worked in direct patient care while sick, respectively.	6/8
Fujino et al. (33)	Japan December 2020 and December 2021	To examine the association between presenteeism and the risk of divorce among Japanese workers during the COVID-19 pandemic	Cohort study	27,036 Participants, with 18,560 in the follow-up	- WFun	Poor marital relationship may have affected presenteeism at baseline. Compared with the group with the lowest WFun score, the OR for the group with moderate WFun was 1.16 (95% CI, 0.74–1.82; *p* = 0.525), and the OR for the group with the highest WFun was 1.76 (95% CI, 1.18 to 2.62; *p* = 0.006).	9/11
Gnanapragasam et al. (39)	United Kingdom March–June 2021	To determine the effectiveness of the ‘Foundations’ application (app) on general (non-psychotic) psychiatric morbidity.	Randomised controlled trial	1,002 HCWs at 16 NHS trusts (multicentre)	Measures were assessed at baseline, after 4 and 8 weeks - GHQ-12 2nd outcomes: BRS-6, SWEMWBS-7; SPS-6; GAD-7; PHQ-9, WSAS-5, MISS-3, and stressors	There was no association between the app group and BRS (aMD = 0.03, 95% CI −0.03–0.09); presenteeism (SPS-6, aMD = 0.38, 95% CI −0.12–0.87); moderate anxiety (GAD-7, aOR = 0.69, 95% CI 0.39 to 01.23); moderate depression (PHQ-9, aOR = 0.61, 95% CI–1.04); moderately severe or severe functioning impairment (WSAS, aOR = 0.61, 95% CI–1.11).	12/13
Hähnle et al. (47)	Germany November 2020 to May 2021 (Psychiatric hospitals)	To examine the consequences of burnout symptoms at the institutional level, such as staff turnover	Cross-sectional study	172 Professionals in Psychiatric hospitals of Children and Adolescents	- BOSS 2nd outcomes: Intention to make shifts, sickness absence in the last 12 months and quality of job performance.	The results show that signs of burnout symptoms impact the turnover tendency, presenteeism, and job performance of professionals. In addition, evidence emerged that professionals were more stressed during the winter lockdown (2020/2021) of the COVID-19 pandemic, and that this influenced turnover tendency, presenteeism, and absenteeism as well as the quality of job performance.	6/8
Jia et al. (44)	China Jane 2020 (Hospital)	To evaluate the direct effects of work stress, health status and presenteeism on task performance, and further explore the mediating effects of health status and presenteeism, hoping to provide theoretical basis for improving the performance of medical staff.	Cross-sectional study	4,261 Medical staff	- CHSS - SF-36 - SPS-6 - Task Performance Scale	The mean scores for work stress, health status, presenteeism and task performance were 2.05 ± 0.84, 4.18 ± 0.68, 2.15 ± 0.79 and 4.49 ± 0.64, respectively. There were significant differences in the task performance scores between different genders, ages, marital statuses, professional titles, departments and work years (*p* < 0.05). Work stress (*β* = −0.136, *p* < 0.001) and presenteeism (*β* = −0.171, *p* < 0.001) were negative predictors of task performance. Health status (*β* = −0.070; *p* < −0.001) and presenteeism (*β* = −0.064; *p* < 0.001) mediated the relationship between work stress and task performance (*p* < 0.001). Presenteeism mediated the relationship between health status and task performance (*β* = 0.07; *p* < 0.001).	8/8
Lee et al. (48)	Republic of Korea 2020	To examine the association between sickness presenteeism and depression among Korean workers during the COVID-19 pandemic in relation with the socioeconomic and lifestyle factors.	Cross-sectional study	Employee group (n = 64,666) and employers or self-employed workers group (n = 19,848).	Korean Community Health Survey - PHQ-9 2nd outcome: sickness presenteeism	Employees in sickness presenteeism showed a higher association with depressive symptoms than employers or self-employed individuals (OR = 2.18, 95% CI: 1.85, 2.56 among employees vs. OR = 1.76, 95% CI: 1.29, 2.40 among employers or self-employed individuals).	8/8
Li et al. (45)	China December 2020 to May 2021 (Hospital)	To investigate the serial-multiple mediating effect of job burnout and fatigue in the relationship between sickness presenteeism and productivity loss among nurses.	Cross-sectional study	2,968 Nurses (multicentre, 14 hospitals)	- Sickness Presenteeism Questionnaire - SPS-6 - Chalder Fatigue Scale - MBI	Sickness presenteeism exhibited a prevalence of 70.6% during the COVID-19 pandemic. The mean score of health-related productivity loss was 15.05 ± 4.52, fatigue was 8.48 ± 3.40, and job burnout was 39.14 ± 19.64. Sickness presenteeism was positively associated with fatigue and job burnout while job burnout was positively associated with nurse fatigue. Sickness presenteeism, fatigue, and job burnout were also positively correlated with health-related productivity loss.	8/8
Mansour et al. (24)	Canada Time 1 October–November 2020, and Time 2 June–July 2021	To examine the role psychosocial safety climate plays as driver or moderator to reduce presenteeism by lessening work intensification over time and the impact of work intensification over time on presenteeism during the COVID-19 pandemic	Cross-sectional study	800 Nurses at Time 1 and 344 at Time 2	- JDS - SPS-6 2nd outcomes: Psychosocial safety climate	Psychosocial safety climate reduces presenteeism over time by reducing work intensification at time 1. Psychosocial safety climate moderates the relationship between work intensification at time 1 and work intensification at time 2. Psychosocial safety climate as moderator also lessens the detrimental effect of work intensification at time 2 on presenteeism at time 2. Presenteeism among nurses affects their health and psychological well-being.	8/8
Nakai et al. (34)	Japan March 2021	To evaluate the impact of the COVID-19 pandemic on employment status, work productivity, QOL, and depressive symptoms in undiagnosed adults with and without attention-deficit/hyperactivity disorder symptoms in Japan	Cross-sectional study	Participants with (*N* = 949) and without (*N* = 942) attention-deficit/hyperactivity disorder symptoms	Japanese Medilead Healthcare Panel - EuroQol 5D-5L - WPAI - PHQ-9 2nd outcomes: Unemployment rate and depressive symptoms	The percentage of impairment with respect to presenteeism was higher in those subjects with ADHD symptoms before the pandemic and without ADHD symptoms before the pandemic than in those with ADHD symptoms during the pandemic and without ADHD symptoms during the pandemic.	6/8
Okawara et al. (35)	Japan December 2020	To examine the relationship between interruption to routine medical care during the COVID-19 pandemic and sickness presenteeism among workers in Japan.	Cross-sectional study	27,036 Workers	CORoNaWork Treatment status, sickness presenteeism and other covariates	The aOR of sickness presenteeism was significantly higher among workers who experienced interrupted medical care (3.44; 95% CI: 3.04–3.89) than among those who did not require routine medical care. The highest OR of sickness presenteeism days was observed for mental health symptoms (aOR: 5.35, 95% CI: 4.85–5.91, *p* < 0.001). When the analysis was performed based on the 36 treatment-symptom groups (3 treatment statuses and 12 symptoms), the largest predictive margin of sickness presenteeism days was observed for mental health symptoms and interrupted medical care (predictive margin: 9.9 days, SE = 0.38)	6/8
Pasfield et al. (50)	New South Wales March–June 2021	To evaluate factors associated with sickness presenteeism in New South Wales registered veterinarians suffering from influenza-like illness, both before and since the beginning of the COVID-19 pandemic	Cross-sectional study	122 Veterinarians	A mixed-methods questionnaire with eight subsections	‘Having no one to cover’ and geographical distribution were significantly associated with sickness presenteeism. Although sickness presenteeism remained common, participants reported that they were less likely to attend work with symptoms of influenza-like illness since the beginning of the COVID-19 pandemic.	8/8
Sagui-Henson et al. (42)	United States March 2020 and March 2021	To examine the effectiveness of evidence-based telecoaching delivered *via* videoconferencing to people requesting mental health services during the COVID-19 pandemic.	Cross-sectional study	1,228 Workers who utilised telecoaching	- WHO-5 well-being questionnaire 2nd outcomes: Burnout, Absenteeism and presenteeism Visit utilisation, and Satisfaction with care	Well-being (*p* = 0.02) significantly increased, while both presenteeism (*p* < 0.001) and absenteeism (*p* < 0.001) significantly decreased at follow-up in the full sample, but represented negligible effect sizes. For every 1 unit increase in the moderator, there was a 0.08-point decrease in presenteeism. When participants completed 1 visit, their presenteeism did not change; when participants completed 2–3 visits, their presenteeism significantly decreased by 0.11 points; and when participants completed 4+ visits, their presenteeism significantly decreased by 0.20 points.	8/8
Schulze et al. (46)	Germany August–October 2020 (nursing homes)	To investigate which psychosocial burdens and potential positive aspects nurses working in long-term care facilities experience during the COVID-19 pandemic	Cross-sectional study	177 Nurses and nursing assistants (nursing homes)	A mixed-methods study - COPSOQ III	The sample scored significantly worse regarding the scales ‘quantitative demands’, ‘hiding emotions’, ‘work-privacy conflicts’, ‘role conflicts’, ‘quality of leadership’, ‘support at work’, ‘recognition’, ‘physical demands’, ‘intention to leave profession’, ‘burnout’, ‘presenteeism’ and ‘inability to relax’. The interviews (n = 15) revealed six main themes related to nurses’ psychosocial stress: ‘overall working conditions’, ‘concern for residents’, ‘management of relatives’, ‘inability to provide terminal care’, ‘tensions between being infected and infecting others’ and ‘technicisation of care’.	6/8
Žilinskas et al. (52)	Lithuania February–April 2021	To conduct an anonymous online survey among white-collar workers from various finance, IT and technology companies in Lithuania to define factors associated with worse sleep quality and diminished productivity during a COVID-19 lockdown.	Cross-sectional study	114 Administrative staff	- PSQI - SLOC - GAD-7 - WHO-HPQ 2nd outcomes: sleep hygiene, physical activity and alcohol use	There was no association between measures of either presenteeism, absenteeism, or sleep locus of control, and general sleep quality (*p* > 0.05). However, there was no strong relationship between sleep-related variables (i.e., sleep hygiene, sleep locus of control, quality of sleep) or levels of anxiety and measures of work productivity.	6/8
Adisa et al. (37)	United Kingdom July–September 2020 (Remote)	To explore how remote working inhibits employee engagement	Qualitative research	32 Workers working from home	Conservation of resources theory Semi-structured interviews	The transition from in-person to online modes of working during the pandemic brought about work intensification, online presenteeism, employment insecurity and poor adaptation to new ways of working from home. These stress factors are capable of depleting vital social and personal resources, thereby impacting negatively on workers engagement levels.	10/11
Ferreira et al. (53)	Portugal April–June 2020 (school and high school)	(1) To understand whether observing cyberbullying among students can be associated with teachers’ productivity loss due to presenteeism and burnout; (2) to examine the role of productivity loss due to presenteeism in the relationship between observing cyberbullying situations among students and teacher burnout.	Cross-sectional study	1,044 Middle school and high school teachers	- Cyberbullying Inventory - SPS - Copenhagen Burnout Inventory Questionnaire	Teacher’s productivity loss due to presenteeism mediated the relationship between observing cyberbullying incidents among their students and their burnout levels. Specifically, the total effect of productivity loss due to presenteeism on teachers’ burnout was 0.57 [CI90, LO = 0.53 HI = 0.62].	8/8
Han et al. (49)	Republic of Korea August 2020	To find predictors of mental health for public health doctors from working experiences at frontline of COVID-19 pandemic.	Cross-sectional study	350 Public health doctors	- PHQ-9 - GAD-7 - PSS - SPS-6	Public health doctors with lowered self-efficacy at work or those exhibiting presenteeism (SPS-6 total score ≥ 19) felt more stress during COVID-19 duty compared to other assignments (AOR = 4.58, 95% CI = 2.32–9.93, *p* < 0.001); a willingness to further volunteer for COVID-19 dispatch was associated with lower odds of presenteeism (AOR = 0.47, 95% CI = 0.26–0.82, *p* = 0.009).	8/8
Hunter et al. (54)	Australia and New Zealand June–August 2020	To determine the associations between health behaviours and work ability and performance during COVID-19 restrictions and if health behaviours were related to demographic or population factors.	Cross-sectional study	433 Adult workers.	- IPAQ - Work Ability Index - WHO-HPQ	A 10% increase in daily sedentary behaviour was associated with 3.68% higher median presenteeism (95% CI: 1.24–6.12%; *p* = 0.003). Being sufficiently physically active was associated with higher reported physical (aOR = 2.1; p = 0.001) and mental work abilities (aOR = 1.8; *p* = 0.007) and self-reported job performance (i.e., lower presenteeism) (median + 7.42%; *p* = 0.03). Part-time workers were 56% less likely (*p* = 0.002) to report a good or very good mental work ability.	8/8
Shimura et al. (36)	Japan 2019 and 2020	To provide empirical evidence of the implications for people and organisations of this new scenario of working from home.	Cross-sectional study	3,123 Office workers from 23 tertiary industries	- BJSQ-57 - PSQI-18 - WLQ-4	5 days a week of remote work (full-remote) was a significant factor for worsening presenteeism (aOR = 1.421, *p* = 0.017) with the adjustment of increasing job stressors (aOR = 1.036/pt., *p* < 0.001), reduced social support (aOR = 1.033/pt., *p* < 0.001), worsening of psychological and physical stress responses (aOR = 1.049/pt., *p* < 0.001), and worsening of sleep disturbance (PSQI) (aOR = 1.080/pt., *p* < 0.001).	8/8
Tilchin et al. (43)	United States March 2020	To understand barriers to staying home from work when sick from COVID-19 (COVID-19 presenteeism) to understand COVID-19 health disparities and transmission and guide workplace and social policy.	Cross-sectional study	220 Workers who worked away from home	COVID-19 presenteeism	Overall, 34.5% of participants reported intended COVID-19 presenteeism. As compared with a salary of less than $35,000, individuals who made $35,000 to $90,000 and individuals who made more than $90,000 had 51% (*p* = 0.033) and 80% (*p* = 0.002) lower odds of COVID-19 presenteeism, respectively. Individuals with insurance versus no insurance had 56% lower odds of COVID-19 presenteeism (*p* = 0.034), individuals who were worried about having enough food versus not worried had 314% higher odds of COVID-19 presenteeism (*p* < 0.001).	6/8
Van Ballegooijen et al. (55)	Belgium and the Netherlands May 2020	To describe: (1) stress, concerns and quality of life; (2) access to healthcare and cancelled/delayed healthcare; and (3) productivity during the first 8 weeks of the coronavirus lockdown in the general population.	Cross-sectional study	2099 Belgian and 2058 Dutch	- VAS (health status) - EuroQol 5D-5L - iMCQ - iPCQ	Productivity losses due to the COVID-19 restrictions were calculated in absenteeism (36%) and presenteeism (30%) for Belgium, and (19%) and (35%) for the Netherlands. Most concerns and productivity losses were reported by respondents with children <12 years, respondents aged 18–35 and respondents with an (expected) COVID-19 infection. The mean value of lost production among respondents in paid profession per person per week including absenteeism and presenteeism was €161.39 for Belgium and €82.69 for the Netherlands.	8/8
Vinberg et al. (18)	Sweden March–April 2021	To analyse the impact of business operations, work and family circumstances, and well-being on the risk of sickness presenteeism for Swedish self-employed workers during the COVID-19 pandemic.	Cross-sectional study	845 Self-employed workers	EQLS and EWCS Outcomes: Sickness presenteeism, Impact on business operations, Risk of bankruptcy, Loss of contracts, Job satisfaction, The index for Work–family conflict, and WHO-HPQ	The impact on business operations (OR = 1.74), loss of contract (OR = 1.41), risk of bankruptcy (OR = 1.15), increase in work hours (OR = 1.41), work–family conflict (OR = 1.45), and mental well-being (OR = 0.86) were significantly related to a higher risk of sickness presenteeism. There was no significant relationship between sickness presenteeism and age, gender, education of the self-employed worker, and company size.	8/8
Blake et al. (38)	United Kingdom April–August 2020 (hospital)	To determine facility usage and gather insight into worker wellbeing and the views of workers towards this provision.	Cross-sectional study	819 Hospital workers.	17-week service use monitoring - SWEMWBS 2nd outcomes: Job stressfulness, job satisfaction turnover intentions, presenteeism, and UWES-9	There was moderate-to-high job stress (62.9%), low wellbeing (26.1%), presenteeism (68%), and intentions to leave (31.6%). There were no significant differences in perceived job stressfulness, job satisfaction, and presenteeism or turnover intentions between those who did, or did not, access a centre.	8/8
Van Der Feltz-Cornelis et al. (40)	United Kingdom May-June 2020 (university, remote)	To explore how the COVID-19 outbreak and arrangements such as remote working and furlough affect work or study stress levels and functioning in staff and students at the University of York, United Kingdom	Cross-sectional study	1,055 University staff and 925 University students	- VAS-scale - PSQ - GAD-7 - PHQ-9 - PHQ-15 - iPCQ	26% of staff and 40% of the students experienced presenteeism. For staff, a model of six variables predicted presenteeism [χ2(6) = 68.40; *p* < 0.001]. Predictors of presenteeism are younger age [OR = 0.97; CI (95) = 0.96–0.98], living with a somatic chronic medical condition [OR = 1.34; CI (95) = 1.03–1.74] or a functional somatic syndrome [OR = 2.14; CI (95) = 1.21–3.80], social isolation [OR = 1.53; CI (95) = 1.05–2.23], no access to outdoor space at home [OR = 1.26; CI (95) = 1.04–1.55], and low current exercise level [OR = 0.78; CI (95) = 0.69–0.89]. Presenteeism was significantly lower in resilient staff (*p* < 0.001)	8/8

aOR, adjusted odds ratio; BDI-2, Beck Depression Inventory-II; BJSQ, Brief Job Stress Questionnaire; BOSS, Burnout Screening Scale; BRS, Brief Resilience Scale; CFT, Cognitive Flexibility Test; CHSS, Challenge-and Hindrance-Related Self-Reported Stress Measures; CI, confidence interval; COPSOQ III, Copenhagen Psychosocial Questionnaire; CORoNaWork, Collaborative Online Research on the Novel-Coronavirus and Work project; EQLS, Eurofound’s European Quality of Life Survey; EWCS, European Working Conditions Survey; GAD, Generalised Anxiety Disorder; GHQ, General Health Questionnaire; HCWs, Healthcare Workers; iMCQ, Medical Consumption Questionnaire; IPAQ, International Physical Activity Questionnaire; iPCQ, iMTA Productivity Cost Questionnaire; JDS, Job Demands Scale; MISS, Minimal Insomnia Symptom Scale; OR, odds ratio; PHQ, Patient Health Questionnaire; PSQI, Pittsburgh Sleep Quality Index and Sleep Schedules; QOL, Quality of Life; MBI, Maslach Burnout Inventory; SF-36, Short Form-36 Health Survey; SLOC, Sleep Locus of Control; SPS, Stanford Presenteeism Scale; SWEMWBS, Short Warwick-Edinburgh Mental Well-being Scale; UWES, Utrecht Work Engagement Scale; VAS, Visual Analogue Scale; WFun, Work Functioning Impairment Scale; WHO-HPQ, World Health Organisation’s Health and Work Performance Questionnaire; WLQ, Work Limitations Questionnaire; WPAI, Work Productivity and Activity Impairment scale; WSAS, Work and Social Adjustment Scale.

## 4. Discussion

The aim of this study was to assess the factors influencing presenteeism or sickness presenteeism and mental health of workers during the COVID-19 pandemic. In this sense, a series of factors related to mental health that may affect presenteeism have been found, as well as a number of factors specific to the individual, factors inherent to the situation caused by the COVID-19 pandemic, and factors derived from working conditions, among others.

### 4.1. Presenteeism and mental health

Stress is one of the main contributing factors to working despite being ill which, in turn, may be one of the reasons why workers continue to work despite being ill (33–35, 38, 42, 44, 45, 47, 49), and in many cases workload, pressure from colleagues, and organisational culture play a part in this relationship (54). Stress was already related to sickness presenteeism prior to the COVID-19 pandemic, so it appears that COVID-19 is not the only factor that may influence sickness presenteeism as expected (55).

There are some high-pressure work environments, such as that of the study by Jia et al. (42), carried out on a sample of 4,261 medical staff, in which it was observed that in high-pressure environments, health problems are more likely to appear and medical staff are more likely to ignore their own health problems, thus increasing sickness presenteeism. In addition to the field of healthcare, it has been observed that the shift from face-to-face work to teleworking has led to workers being forced to be constantly online and on email, thereby generating constant stress, as they worry about losing their jobs (35). For many workers, they had to stay online at all times to prove their worth at work or to convince their employers that they were not avoiding their duties while working from home (56).

Continuous stressful situations can lead to sickness presenteeism due to burnout, as observed in the studies by Basar et al. (49), Ferreira et al. (51), Haehnle et al. (45), and Shimura et al. (34), and to symptoms of depression as seen in the study by Lee et al. (46), in which an association between SP and depression was found to be higher among blue-collar and less educated workers. In the case of the latter, the depressive symptoms of workers who were not able to obtain paid sick leave were 2.18 times higher than those who had the option to do so, hence symptoms of stress, depression, or anxiety were likely to appear. These excessive work demands may lead to presenteeism, while burnout may be a consequence resulting from this situation (9). In this regard, there are a number of factors that could buffer these demands, such as well-being (16, 40) or work engagement (55).

On the other hand, there was only one study that determined a relationship between nurses’ fear of contracting COVID-19 and stress-related presenteeism (49), which can lead to reduced performance, productivity, and efficiency in organisations (57). Other factors such as cyberbullying (51), sleep disturbance (34), concern about having enough food (41), social isolation (38), and no resilience (38) were also related to sickness presenteeism.

### 4.2. Presenteeism and individual factors

It was observed that having a chronic illness could be correlated with sickness presenteeism despite having a decompensated disease. In fact, at the onset of the disease, workers continue to work despite manifesting symptoms until they are forced to take sick leave due to exacerbation of the symptoms or prolonged duration of the disease (38). To avoid this problem, continued regular treatment is recommended in order to manage the disease and maintain health (56), as was the case among workers who experienced interrupted medical care (33, 40).

On the other hand, self-perception of one’s own health status determines whether workers assess their illness as sufficiently serious, moderate or mild for them to continue working or not (42). It is known that when working in high-pressure environments, health problems are more likely to occur and therefore, health is compromised (58). In this case, during the COVID-19 pandemic, fever was identified as one of the main symptoms used by workers to be absent from work as it may be related to COVID-19 (48). However, previously, this type of symptom was not a usual reason for taking sick leave and some workers, despite having fever, continued to work. In fact, feeling unable to take sick leave can negatively affect health and vice versa (59).

Other factors such as poor marital relationship (31), being young (38), attention-deficit/hyperactivity disorder symptoms (32), low physical activity (38, 50) and sedentary behaviours (52), having children (41), and having health insurance (41) may be related to sickness presenteeism.

### 4.3. Presenteeism and factors related to the situation caused by the COVID-19 pandemic

In a study conducted in New South Wales between March and June 2021 on a sample of 122 veterinarians, it was determined that one of the factors associated with sickness presenteeism among those suffering from influenza-like illness during the COVID-19 pandemic was that they attended work despite having symptoms of respiratory infectious disease (48). The same could happen with COVID-19 signs and symptoms; people with mild symptoms may continue attending work despite the possible risk of virus transmission (57). This could again be explained by the sample’s high level of work engagement, the shortage of staff, and the company’s specific sick leave policies (36). According to Okawara et al. (33), workers do not attach the same importance to some signs and symptoms as to others. Those symptoms related to mental health, pain, burnout, or sleep were more susceptible to higher sickness presenteeism, whereas others such as signs and symptoms related to skin or hair problems, etc. showed moderate levels of sickness presenteeism and workers did see the need to take sick leave due to this type of symptomatology. This dichotomy will depend on the individual and whether they consider the symptoms to be sufficiently adverse or severe (60). In this regard, consideration should be given to what is meant by ‘unable to work due to illness’, i.e., is it a total inability to work, or is it an inability to perform functions at an expected level? (48).

Other factors contributing to presenteeism or sickness presenteeism during the COVID-19 pandemic (16) may be its impact on business operations, loss of contract and risk of bankruptcy, not volunteering to work on the front line (47), or confinement itself (45), which may in turn be indicators of poor socio-economic and working conditions (54).

### 4.4. Presenteeism and factors related to working conditions

Direct patient care (39) and workload (22) may be factors associated with presenteeism, which is particularly observed in services with a shortage of staff and with workers under high time pressure (5). Related to the above, perceived organisational support (49) and safety climate (22) may be contributing factors to sickness presenteeism. In some organisations, it is not easy for workers to choose to stay at home when they are sick, which may lead to frustration, resentment towards the company, depressive symptoms, and lower work engagement (61).

Only one of the studies (45) analysed the relationship between shift work and sickness presenteeism. As in other studies conducted prior to the COVID-19 pandemic (62), health workers who were on shifts attended work while sick more often than health workers who were not on shifts, and perhaps this may be influenced by their own biorhythms. Work-related fatigue may also be related to sickness presenteeism (34, 43), so long working hours need to be managed (62), communication and monitoring systems within the company should be improved, and a replacement plan should be in place to prevent workers from not taking sick leave on the grounds that there is no one to cover them (48).

Regarding the transition from in-person to online modes of working (34, 35), improving the work environment for workers while working from home is important to reduce the negative health outcomes associated with this type of activity, reduce absenteeism, and increase productivity.

Other variables related to working conditions may be work functioning or task performance impairment (31, 42, 43, 53), salary of less than $35,000 (41), increased working hours, work–family conflict (16), and geographical distribution (48).

It is estimated that the mean value of lost production per person per week, including absenteeism and presenteeism, can be in a range between €161 and €82 (53).

Contrary to many studies, there was one study in which the authors found no significant relationship between sickness presenteeism and age, sex, education of the self-employed, and size of the company (16). This could be explained by the characteristics of the sample, being young and highly engaged workers.

Finally, in a meta-analysis that assessed the status and factors influencing presenteeism among clinical nurses before the pandemic (63), it was observed that presenteeism scores were higher in publications prior to 2020, but in this case, they did find statistically significant differences in terms of age, sex, marital status, experience, region, and service groups that could be explained by the change in working conditions that a pandemic such as the COVID-19 one has brought about. In this line, and as has been detected, sickness presenteeism has been found to be a risk factor for future sickness absenteeism and may lead to decreased self-perceived health as observed in a systematic review conducted prior to the COVID-19 pandemic (64).

### 4.5. Limitations

There are a number of limitations to this study. Although certain factors favouring or reducing the likelihood of sickness presenteeism have been detailed, it is possible that many of these factors are a consequence of sickness presenteeism or may even interact with it, and it might not be possible to discern cause from consequence. On the other hand, the samples were highly heterogeneous, and the time of collection and the instruments used also differed, making it difficult to compare the samples, which is why no meta-analysis was proposed. Most of the finally selected studies were cross-sectional and used hetero-administered instruments *via* online surveys, with the limitations that this method entails. Finally, each country has its own rules on sick leave entitlement, which may result in a person needing to continue to work despite being ill.

## 5. Conclusion

A number of factors have been identified that influence mental health and sickness presenteeism, such as factors directly related to mental health (burnout, stress, depression, fear of COVID-19, no well-being, cyberbullying, sleep disturbance, concern about having enough food, social isolation, and no resilience); individual factors (poor marital relationship, health status, being young, attention-deficit/hyperactivity disorder symptoms, workers who experienced interrupted medical care, low physical activity and sedentary behaviours, having children, having health insurance, and having a chronic illness); factors related to the situation caused by the COVID-19 pandemic (confinement, having symptoms of respiratory infectious disease, not volunteering to work on the front line, impact on business operations, loss of contract, and risk of bankruptcy); and factors arising from working conditions (perceived organisational support, direct patient care, work functioning impairment or task performance, work fatigue, safety climate, workload, having no one to cover them, geographical distribution, transition from in-person to online modes of working, salary of less than $35,000, increased working hours, and work–family conflict).

Identifying the key drivers of presenteeism or sickness presenteeism and understanding the underlying phenomena and origins will help to create a safe working environment and optimal organisational systems to protect vulnerable workers from medical and occupational adversity, especially in a pandemic context where changes, challenges, and consequences have had a considerable impact.

## Data availability statement

The original contributions presented in the study are included in the article/Supplementary material, further inquiries can be directed to the corresponding author.

## Author contributions

JJG-I, JG-S, JA, RR, EC, CR-F, SM-I, DF-G, and ÁV-A: conceptualization, data curation, formal analysis, investigation, methodology, resources, software, supervision, validation, visualization, writing – original draft, and writing – review and editing. JJG-I, JG-S, JA, RR, EC, and ÁV-A: project administration. All authors contributed to the article and approved the submitted version.

## Conflict of interest

The authors declare that the research was conducted in the absence of any commercial or financial relationships that could be construed as a potential conflict of interest.

## Publisher’s note

All claims expressed in this article are solely those of the authors and do not necessarily represent those of their affiliated organizations, or those of the publisher, the editors and the reviewers. Any product that may be evaluated in this article, or claim that may be made by its manufacturer, is not guaranteed or endorsed by the publisher.
