# Supplementary material for: Presenteeism and mental health of workers during the COVID-19 pandemic: a systematic review
Source: Front Public Health. 2023 Sep 14;11:1224332. doi: 10.3389/fpubh.2023.1224332 (PMC10536966; doi:10.3389/fpubh.2023.1224332)
Supplement: Supplementary file 1 [file Table_1.docx]

**SUPPLEMENTARY MATERIAL**

**Table S1.** Scores of analytical cross-sectional studies

| **Studies** | **JBI** | **The participants and the environment are described in detail** | **Inclusion criteria are clearly defined** | **Exposure was validly and reliably measured** | **The criterion used to measure the condition was objective** | **Confounding factors were identified** | **Strategies for dealing with confounding factors** | **Results measured in a valid and reliable way** | **Appropriate statistical analysis was used** |
| --- | --- | --- | --- | --- | --- | --- | --- | --- | --- |
| Basar et al., 2022(51) | 8/8 | ☺ | ☺ | ☺ | ☺ | ☺ | ☺ | ☺ | ☺ |
| Cheslack-Postava et al., 2022(41) | 6/8 | ☺ | ☺ | ☺ | ☺ | ☹ | ☹ | ☺ | ☺ |
| Hähnle et al., 2022(47) | 6/8 | ☺ | ☺ | ☺ | ☺ | ☹ | ☹ | ☺ | ☺ |
| Jia et al., 2022(44) | 8/8 | ☺ | ☺ | ☺ | ☺ | ☺ | ☺ | ☺ | ☺ |
| Lee et al., 2022(48) | 8/8 | ☺ | ☺ | ☺ | ☺ | ☺ | ☺ | ☺ | ☺ |
| Li et al., 2022(45) | 8/8 | ☺ | ☺ | ☺ | ☺ | ☺ | ☺ | ☺ | ☺ |
| Mansour et al., 2022(24) | 8/8 | ☺ | ☺ | ☺ | ☺ | ☺ | ☺ | ☺ | ☺ |
| Nakai et al., 2022(34) | 6/8 | ☺ | ☺ | ☺ | ☺ | ☹ | ☹ | ☺ | ☺ |
| Okawara et al., 2022(35) | 6/8 | ☺ | ☺ | ☺ | ☺ | ☹ | ☹ | ☺ | ☺ |
| Pasfield et al., 2022(50) | 8/8 | ☺ | ☺ | ☺ | ☺ | ☺ | ☺ | ☺ | ☺ |
| Sagui-Henson et al., 2022(42) | 8/8 | ☺ | ☺ | ☺ | ☺ | ☺ | ☺ | ☺ | ☺ |
| Schulze et al., 2022(46) | 6/8 | ☺ | ☺ | ☺ | ☺ | ☹ | ☹ | ☺ | ☺ |
| Žilinskas et al., 2022(52) | 6/8 | ☺ | ☺ | ☺ | ☺ | ☹ | ☹ | ☺ | ☺ |
| Ferreira et al., 2021(53) | 8/8 | ☺ | ☺ | ☺ | ☺ | ☺ | ☺ | ☺ | ☺ |
| Han et al., 2021(49) | 8/8 | ☺ | ☺ | ☺ | ☺ | ☺ | ☺ | ☺ | ☺ |
| Hunter et al., 2021(54) | 8/8 | ☺ | ☺ | ☺ | ☺ | ☺ | ☺ | ☺ | ☺ |
| Shimura et al., 2021(36) | 8/8 | ☺ | ☺ | ☺ | ☺ | ☺ | ☺ | ☺ | ☺ |
| Tilchin et al., 2021(43) | 6/8 | ☺ | ☺ | ☺ | ☺ | ☹ | ☹ | ☺ | ☺ |
| Van Ballegooijen et al., 2021(55) | 8/8 | ☺ | ☺ | ☺ | ☺ | ☺ | ☺ | ☺ | ☺ |
| Vinberg et al., 2021(18) | 8/8 | ☺ | ☺ | ☺ | ☺ | ☺ | ☺ | ☺ | ☺ |
| Blake et al., 2020(38) | 8/8 | ☺ | ☺ | ☺ | ☺ | ☺ | ☺ | ☺ | ☺ |
| Van Der Feltz-Cornelis et al., 2020(40) | 8/8 | ☺ | ☺ | ☺ | ☺ | ☺ | ☺ | ☺ | ☺ |
|  |  |  |  |  |  |  |  |  |  |

Yes: ☺, No: ☹, Not clear or Not Applicable: 😐

**Table S2.** Scores of qualitative research ~~(~~

| **Studies** | **JBI** | **Congruence between stated philosophical perspective and research methodology** | **Congruence between research methodology and research question/objectives** | **Congruence between research methodology and methods used to collect data** | **Congruence between research methodology and representation and analysis of data** | **Congruence between research methodology and interpretation of results** | **Cultural and theoretical localisation** | **Influence of the researcher on the research and vice versa is addressed** | **Representation of participants and their voices** | **Ethical approval by an appropriate body** | **Relationship between findings and data analysis or data interpretation** |
| --- | --- | --- | --- | --- | --- | --- | --- | --- | --- | --- | --- |
| Adisa et al., 2021(37) | 10/10 | ☺ | ☺ | ☺ | ☺ | ☺ | ☺ | ☺ | ☺ | ☺ | ☺ |

Yes: ☺, No: ☹, Not clear or Not Applicable: 😐

**Table S3.** Scores of randomised controlled trials

| **Studies** | **JBI** | **True randomisation used for assignment of participants to treatment groups** | **Allocation to treatment groups concealed** | **Treatment groups similar at the baseline** | **Participants blinded to treatment assignment** | **Those delivering treatment blinded to treatment assignment** | **Outcomes assessors blinded to treatment assignment** | **Treatment groups treated identically other than the intervention of interest** | **Follow-up complete and if not, differences between groups in terms of their follow-up adequately described and analysed** | **Participants analysed in the groups to which they were randomised** | **Outcomes measured in the same way for treatment groups** | **Outcomes measured in a reliable way** | **Appropriate statistical analysis used** | **Trial design appropriate and any deviations from the standard RCT design accounted for** |
| --- | --- | --- | --- | --- | --- | --- | --- | --- | --- | --- | --- | --- | --- | --- |
| Gnanapragasam et al., 2022(39) | 12/13 | ☺ | ☺ | ☺ | ☹ | ☺ | ☺ | ☺ | ☺ | ☺ | ☺ | ☺ | ☺ | ☺ |

Yes: ☺, No: ☹, Not clear or Not Applicable: 😐

**Table S4.** Scores of cohort studies

| **Studies** | **JBI** | **Two similar groups and recruited from the same population** | **Exposures measured similarly to assign people to both exposed and**  **unexposed groups** | **Exposure measured in a valid and reliable way** | **Confounding factors identified** | **Strategies to deal with confounding factors stated** | **Groups/participants free of the outcome at the start of the study (or at the moment of exposure)** | **Outcomes measured in a valid and reliable way** | **Follow-up time reported and sufficient to be long enough for outcomes to occur** | **Follow-up complete, and if not, reasons to loss of follow-up described and explored** | **Strategies to address incomplete follow-up utilised** | **Appropriate statistical analysis used** |
| --- | --- | --- | --- | --- | --- | --- | --- | --- | --- | --- | --- | --- |
| Fujino et al., 2022(33) | 9/11 | ☺ | ☺ | ☺ | ☺ | ☺ | ☺ | ☺ | ☺ | ☹ | ☹ | ☺ |

Yes: ☺, No: ☹, Not clear or Not Applicable: 😐
